# Extra Supplement—The Nursing Mirror

**Published:** 1890-08-02

**Authors:** 


### "The Hospital" Nursing Mirror.

Being the Extra Nursing Supplement of "The Hospital" Newspaper.

All Contributions for this Supplement should be addressed to the Editor, The Hospital, 140, Strand, London, W.C., and should have the word "Nursing" plainly written in left-hand top corner of the envelope.

### En Passant.

A NEW HOME AT BRIGHTON.—We are glad to see that Miss Steward, trained and certificated lady nurse, has taken 6, Belgrave Place, Brighton, for the reception of medical and surgical patients, and a few convalescents. The sanitary arrangements have been carefully overhauled, and the terms are according to requirements.

A PRAYER SOCIETY.—Miss Ada B. Tyson, of 9, Gayton Crescent, Hampstead, sends us word that she has started a society of Prayer and Help for our Hospitals. The members are to pray for the hospitals and make clothes for the inmates. If Miss Tyson had written on one side of the paper only, we would gladly have published her letter in full.

SHORT ITEMS.—The nurses of the Royal Free Hospital have cake and jam for tea. We should not have thought this worth printing save that late proceedings point to the fact that some probationers think as much about their food as about their patients.—The services of the Cyprus Society nurse have been accepted by Sir H. Bulwer for the Government Hospital, and the appointment of Nurse Christian has met with great satisfaction.—A first congress of monthly nurses, *Erste Deutsche Hebammentag*, is to meet in Berlin, on September 22nd and 23rd, for a discussion of the rights and interests of these ladies.—A Committee is to be appointed, on the motion of Canon Fielden, to consider the establishing of a nurses' home in connection with Norfolk and Norwich Hospital.

BRITISH NURSES' ASSOCIATION.—Miss Foggo Thomson has resigned the secretaryship of the British Nurses' Association, which appointment she assumed in February last. Having regard to the fact that Miss Foggo Thomson was described on her appointment as possessing "an amount of literary talent, shrewd common sense, and practical originality, which will be of inestimable service to the British Nurses' Association," we should like to know the true cause of her resignation. We take this opportunity of correcting an error into which we inadvertently fell in regard to Miss Foggo Thomson. Miss Thomson has been eight years a nurse, and has worked at the Children's Hospital, Birkenhead, at the Hope Hospital, Manchester, and the Royal Hants County Hospital, Portsmouth. She was also engaged in Manchester for over a year on the staff of the Manchester and Salford Sick Poor and Private Nursing Institution.

NURSES.—Under this simple heading, an admirable little article has appeared in many local papers. Says the writer: "If a few of the thousands of men, women, and little children taken helpless to hospital wards were asked for their testimony as to the conduct of the nurses, the chorus of eulogy would be very touching and beautiful to hear. There are, naturally, many failures, especially among the ladies, who scarcely realise what nursing means; but they are but a drop in the great army of brave, competent workers—women whose nerve in the operating theatre is on a par with that of the coolest surgeon, and whose tender pity equals that of the most sensitive of their sisters." The article concludes with a plea for legislation as to the hours of nurses. We think an appeal to hospital committees and training school authorities would be better. They can understand more fully than the House of Commons, the trials of the nurses in hospital wards.

HELP WANTED.—It seems only too certain that no home for epileptic cases exists; a crying need which should be redressed. But here is another case which surely some of our readers can assist us with. A young man, now in the Princess Alice Memorial Hospital, wants to hear of a home where he can be received for a small weekly charge. He is paralysed and incurable; his mother could pay a few shillings weekly for him. The Rev. C. C. Crake, of Polegate, will give any information about the case.

KENT NURSING INSTITUTION.—The very pretty bazaar which Princess Louise opened in Knole Park, in aid of the Kent Nursing Institution, was very successful. It was a highly-fashionable gathering, but the nurses had a stall of their own, and quietly did good work. The institution has homes at Tunbridge Wells and West Malling. There are twenty-seven nurses on the staff, and a few probationers. Princess Louise presented Nurse Pennington and others with the silver medal of the institution.

PARIS INSTITUTIONS.—A Paris paper contains a eulogistic article on the Nursing Institution at 28, Rue des Acacias, established by a Danish lady, Mdlle. Nielson. According to this account, trained nurses of great experience, holding diplomas and speaking several languages, can be obtained there for private cases at a moment's notice. There are also a few French nurses, and patients can be taken into the home. The full title of the home is: "L'Institut Pour Gardes-Malades Diplomées Anglaises et Scandinaves."

THE EIGHT HOURS DAY.—It is so very easy to point out faults in the administration of large institutions, but it is another thing to propose a practical remedy. The Lords' Committee think eight hours is a sufficiently long day for nurses; so do we; but how is it to be arrived at? The proposal that the nurses are to "work in three shifts" fails not only because of want of funds to house and keep, and train so many nurses, but because it would be detrimental to good nursing. It is hard enough now for the patient to be at the mercy of two sets of nurses, one for day and one for night duty, but three sets of nurses would inevitably work confusion. Doctors would not hear of it; nurses would not hear of it. A good nurse will often work night and day rather than leave a private patient during a crisis, and what is more, a doctor will expect this from a nurse. A remedy in hospital work might surely be arrived at by having extra ward maids to do the dusting and sweeping and polishing in the morning, and letting the night nurses go off duty when the day came on. Take the hours of the London nurses as given before the Lords' Committee: Night nurses from 9.30 p.m. to 9.30 a.m.; day nurses from 7 a.m. to 9.30 p.m., with two hours off in the afternoon. From 7 a.m. to 9.30 a.m., then, the two sets of nurses are working together, and this is necessary, because the morning work involves much heavy cleaning and dusting. Could not the night nurses go off duty at 8.30, thus reducing their working hours to eleven, and the day nurses not come on till 8.30 a.m., reducing their working hours to eleven also? From 7 to 9 the servants of the nursing home, or extra ward maids, could sweep and dust under the supervision of the nurses. This would bring us one hour nearer to the ideal, but at present impracticable eight hours' day.

### Sketches of Insanity.

By Francis H. Walmsley, M.D. (Senior Assistant Medical Officer of Leavesden Asylum).

### V.—MANIA.

There are in the brain certain points or areas—known as controlling and harmonizing centres—that regulate and control the cerebral operations. These centres are the seat both of intelligence and of voluntary muscular activity. In a state of mental health there is a peculiar harmony in their action and in their relation to one another. In mania and other forms of insanity the natural, harmonious, concerted action of these different centres is disturbed, there is disharmony and discord; a state of morbid over-activity is induced, consciousness becomes a torrent of intense thoughts and feelings, the patient is governed by despotic emotions that successively depose one another, his behaviour becomes explosive, chaotic, incalculable; muscular activity varying in amount becomes the natural language of feeling varying in amount. As a greater or less number of these centres—mental and motor—are in disharmony, so will there be a greater or less degree of intellectual perversion, emotional excitement, and muscular disturbance.

The characteristic, distinguishing the most perfectly constituted of our race is the altruistic sentiment, or that active fellow-feeling which holds egoism in check and which expends itself in spontaneous efforts to further the welfare of others; conversely in the lunatic the prominent characteristic is a morbid egoism which displays itself in every stage of his malady. A morbid self-feeling is the beginning, and intensified, is the culmination of active and acute mania. The madman lives in himself, he is at war with everyone, his predominant mood is one of antagonism and combat; in his conduct we see the widest departure from the established rules of human action.

In mania there may be displayed every shade of feeling, from gloomy and suspicious irascibility to great hilarity, the irascibility implies a relative inactivity of the superior feelings. For the normal sensation of existence there may be substituted undue joyousness, exuberant emotion, and extreme content; misleading expressions of grave disintegration.

In the early stage of the malady there is such a mental change in the individual as causes him to act not in harmony with his natural self and his surroundings. There is perceptible, to those who know him, an alteration in his habits of thought, feeling, and action; he manifests fitfulness and uncertainty in conduct, is readily excited to anger or laughter, neglects his family, forsakes his business, becomes loquacious, talking constantly about himself and his private affairs; he forms an exaggerated view of his own importance. There may be a consciousness on the part of the patient of some disorder in his mental faculties; he makes great efforts to appear reasonable; he may be more quiet and dull than usual, or, on the contrary, more restless and excitable; he may cry or sing, and appear like a person intoxicated. If we try to convey to such an individual a family trouble or the loss of one formerly loved, if we seek to set some chord of emotion vibrating within him, nothing moves him, his feeling and fine sensibilities are blunted. This stage may continue for a longer or shorter period, passing on into acute mania, the patient becoming wild and agitated, ferocious or gay; confusion reigns in his mind, his ideas follow one another without mutual connection, there is failure of the controlling power of reflection and judgment, the memory is disorganized, vague recollections attain to extraordinary intensity, the patient mistaking remembrances of the past for the realities of every-day life. He has lost the power of taking his bearings as to the position of things about him, he is haunted by wild delusions, which at times take entire possession of him, and under the influence of which he acts in the most extraordinary manner. Owing to the abnormal information conveyed through the muscular sense, maniacs often entertain the belief that they are endowed with superhuman strength, that they can run, fly, lift enormous weights, &c. Their delusions, which are fleeting and various, generally pertain to self, they exult in the belief that they are of great personal attractions, of great power, of exalted rank; or that they are possessed of great inventive faculties. In accordance with his various moods, the maniac commits frivolous or harmless actions, he spends his time—fantastically decorated—in singing, shouting, gesticulating, dancing, running, tearing up and destroying everything within his reach, fighting and committing violent and unprovoked assaults, he is in a state of incessant movement and vociferation. The organs of expression by movement are primarily the features, next the voice, lastly, the movements of the body at large. The grimaces, gestures, and attitudes show an accession of active power. Attention may be drawn to the strong resemblance existing between the phenomena attending a fit of intoxication, or a paroxysm of anger, and the phenomena displayed during an attack of transient mania.

*(To be continued.)*

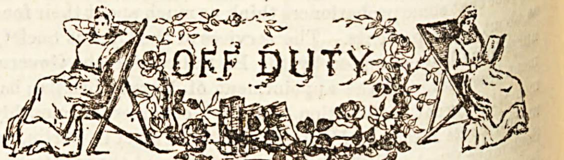

### Dr. Tarrant's Mistake.

Erasmus Tarrant, M.D., F.R.C.S., one of the principal surgeons of St. Botolph's, commonly known as Bott's, was sitting in the common room at that hospital snatching a hurried lunch before proceeding to the operating theatre. He was noted in the medical profession for his minute acquaintance with diseases of the brain, and the peculiar boldness and skill with which he operated on that organ. He had published a work on Craniology, painstaking and accurate in its observed cases, but brilliant and startling in speculation, which at once placed him in the first rank of his fellow specialists, and he had now held for five years the position of surgeon to the chief London Hospital for Diseases of the Brain. He was still a comparatively young man, barely thirty-five, but prematurely grave and serious in manner. He was broad-shouldered and thick-set, of middle height, with a large triangular-shaped head set on his shoulders without any apparent interval of neck. His pale face was clean shaven, but, if he curtailed his beard, he made up for it by letting his shock of dull brown hair grow as Nature prompted it. The man's features were uninteresting and common-place in the extreme; the eyes small and insignificant, the whole physiognomy devoid of expression. His forehead, however, was broad and massive, and the cranium, when seen in profile, swept back with a full and perfect curve, from which a close observer would gather that Dr. Tarrant was not devoid of ability. Although the Doctor was always courteous and obliging to the students, yet he was not popular. He never complained if they shirked his lectures, and always signed everyone up, as a matter of course, but he took no sort of interest in them. "He will do nothing for you, he will give himself no trouble whatever about you, unless he can get something out of you 'for his own advantage.' " He was also accused of being reckless in the matter of operations, of always operating even when the chance of success was very small. This tendency to unjustifiable operations, a tendency most properly condemned by medical men, was attributed to the fact that he had walked the Paris hospitals, for French surgeons, whether rightly or wrongly, are generally considered less scrupulous than their English brethren. Perhaps the hospital did not quite do him justice. It seems to have been true that he took no interest in anything outside his speciality, but he never attempted to induce men to be coached by him, and he was absolutely indifferent as to money. He was always ready to give any amount of assistance to any man who possessed sufficient ability to help him in his own researches. If he did operate over readily, he was attentive and painstaking to a degree which has rarely been equalled by the best surgeons. So far as I have been able to gather the love of science in Dr. Tarrant really did exclude all other affections, and he appears to have regarded mankind more as complex nervous mechanisms than as fellow creatures, but it is quite possible that my informant's judgments were false or one-sided, as they knew nothing of his private life, having only met him in his official capacity.

Scarcely had Dr. Tarrant finished his lunch, when a messenger came hurriedly in to summon him to an urgent case in the accident ward. When he got there he found the patient, a tall lean man of about thirty, lying unconscious on one of the beds, his head swathed in blood-stained bandages. The man's coat and waistcoat had been removed, but he was still wearing the rest of his clothes, which were those of a man in a good position in society. The facts of the case were very simple. A cabman had taken up a fare on Blackfriars Bridge, but before he had driven many yards he was stopped by the report of a revolver, and getting off the box, found his fare had fallen forward in the cab, and was bleeding profusely from a wound in the forehead. Thereupon he bandaged the wound as well as he could with his own and the sufferer's pocket handkerchiefs, and drove off to the nearest hospital, which happened to be St. Botolph's. The surgeon rapidly, but carefully removed the handkerchiefs, extracted a few splinters of bone from the wound with a forceps, and then re-bandaged it again.

"Nothing can be done at present," he said to the two dressers and the matron of the ward, who were standing by. "He must recover from the shock before we can investigate further. Send for me at once if he shows the slightest signs of recovering consciousness."

In reality Dr. Tarrant considered the man as practically dead, for he made the following note in his book :—

Male about 30, name unknown. Bullet wound in the temporal bone, just above frontal sinus, slightly to the left of the medial sinus; bullet lodged probably in left hemisphere. Sinking. No paralysis.

But the stranger did not sink, his breathing was laboured and heavy, and his body was agitated from time to time by violent twitchings, but the heart beat strongly, the temperature was well maintained, and no sign of collapse appeared. In about two hours he recovered partial consciousness and opened his eyes, whereupon the doctor was at once summoned. The patient seemed sensible and in no way delirious, but he had lost the power of co-ordinated speech and motion ; he kept muttering meaningless words and portions of words, clearly knowing what he wished to say but incapable of saying it. His hands kept quivering about with a jerky movement like those of a man suffering from nightmare ; his eyelids were winking and blinking, whilst his eye-balls from time to time were turned up so as only to show the whites. Dr. Tarrant walked up the ward with his quick, noiseless step, looking the picture of official imperturbability, but as soon as he caught sight of the wounded man he first seemed astonished and then puzzled.

*(To be continued.)*

### Everybody's Opinion.

*[Correspondence on all subjects is invited, but we cannot in any way be responsible for the opinions expressed by our correspondents. No communications can be entertained if the name and address of the correspondent is not given, or unless one side of the paper only be written on.]*

### HOMES FOR EPILEPTICS.

We have had some most interesting letters on this subject, and should like to learn the views of some readers as to whether a special home is necessary or not. Miss Louisa Twining writes: As I have for many years been interested in this subject, I may be able to reply to the inquiries of your correspondent in the same terms, as, alas ! hitherto. I do not know of any homes for this large and afflicted class of persons (I especially allude to young women and girls) for less payment than £40 or £50 a-year, the former being the lowest anywhere. Between these and the poor-law infirmaries, or, rather, asylums, there was no available provision—certainly a few years ago. For the upper classes, from £100 to £200 or more is charged. For some years, feeling the great need that existed, I took into a home a few young women, able to pay 10s. week, and our applications far exceeded our space. Governesses, teachers, members of families who could not be kept at home with safety to the younger ones, thankfully availed themselves of what, I believe, was the first and only offer of a home on terms which were possible for them. The subject has often been discussed, and a meeting was held at the rooms of the C.O.S. a few years ago. I then urged what I believe to be the only means of help for this large class of sad sufferers, the opening of a large institution like the Asylums for Lunatics, where payments of 10s. a-week and upwards could be made, and were the building rent free, these would nearly, if not wholly, pay for maintenance, for many of the upper classes would be thankful to avail themselves of such asylums for high payments. Needlework and many other employments would help the expenses, as, under direction, much skill is shown in these ways. Those who have visited Leavesden Asylum for Imbeciles and Epileptics of the pauper class, will agree with me that it is sad such noble institutions, where every comfort and alleviation is provided, should be available for the poorest alone ; while those who are willing and able to help themselves, of the middle-classes, should be left absolutely unaided in this saddest of all afflictions. I earnestly hope and desire to see some consideration given to this most important matter. Even if asylums had to be partly supported by rates, is not the need of the same kind as in the case of lunatic asylums? for mischief at home is caused to many by keeping such patients. There are two vacancies for chronic invalid women in the Mission House, Victoria Road, Worthing, for payments of 12s. a-week. At present there is no help for this class of sufferers (as I believe) in the southern counties of England, and it is hoped that this is only the beginning of a plan to be further developed in the future. Applications may be made to the Sister-in-Charge, St. Andrew's Mission House, Worthing. [See also Answers on next page.]

### NURSES IN ASYLUMS.

Miss Pauline Buckland writes from Berry Wood, Northampton: Will you kindly allow me space in your paper to make a few remarks on a sentence I noticed in this week's issue of The Hospital, viz., "Shall we in time achieve our heart's desire of securing the right of the dying lunatic to be properly nursed?" To me it seems almost incredible that anyone should imagine the insane are neglected in any way; but the above quotation certainly implies that the dying lunatic does not receive necessary attention. I am very sorry to find there is still such a strong prejudice against asylum nurses and the manner in which asylum patients are treated. I cannot speak for other institutions (though I have no doubt that in all provincial asylums every possible care and attention is bestowed on the patients, whether they are in good or ill health), but I do know that it would be impossible for the "dying lunatics to be more properly nursed," in every sense of the word, than they are here. Some time ago two hospital-trained nurses were appointed for the infirmary ward here, but both were failures; both were sent away in disgrace, after very short service, because they did not give proper care and attention to the patients in their charge.

### LOYALTY.

"Poor Pro." writes: Some time ago, Mr. Editor, you said that women were wanting in loyalty and the spirit of *camaraderie.* Well, I have just come back from the Select Committee on Hospitals, and I have never seen a prettier sight than the way the staff and sisters of the London Hospital support their matron during her present trials. There they were, all grouped round her, anxiously pressing forward to shake hands with her, and in every way showing a loyal devotion greatly to the credit of their sex. Mr. Editor, you must never say again that women are not loyal to one another, and cannot work under one of their number in peace! Go and see Miss Lückes with her assistants and her sisters, and you will see women who are true and faithful to one another, and who are unruffled by petty spites and jealousies. You will think better of all women ever afterwards.

### CHARITY ORGANISATION SOCIETY.

Mr. C. S. Loch writes: My attention has been called to a descriptive article, signed "Gregory," in the Nursing Supplement of The Hospital of June 21 last, giving an account of a nurse's sojourn in a lodging-house. In the final paragraph it is stated that "the Charity Organisation Society sells tickets to the charitably-disposed in connection with this Home." I think that your correspondent must be under a misapprehension. Perhaps she would kindly give me the authority for her statement?—[The article referred to a Glasgow lodging-house.—Editor.]

### Notes and Queries.

### Queries.

(26) *Convalescent Home Wanted.*—For a poor man who has an open wound which requires dressing daily. He could afford to pay a little.—*Puss.*

(27) *Montreal.*—Can any nurse tell me anything about Miss Gee's Home at Montreal?—*H. M.*

### Answers.

*B. C. M.*—Unless it distinctly states in the agreement you signed that your wages would be stopped in case of illness, the superintendent has no right to stop them. Nurses should be more careful as to the agreements they sign. The shame of publicity would punish any institution behaving unfairly.

*Puss.*—We never prescribe.

*A Lady Nurse, Winchester.*—It is not worth while slaying the slain. Mrs. Lynn Linton is no authority on nursing.

*T. P.*—The idea is very pretty, but the lines do not scan. You need more experience in verse making.

(15) "*A Home for Epileptics.*"—Hospital for Epilepsy, Paralysis, and other Diseases of the Nervous System, Portland Terrace, Regent's Park, N.W. (near St. John's Wood Road Station). Both in and out patients are expected to pay according to their means, but the necessitous poor receive free treatment. Low's Book of Charities.—*Grace.*

In reply to the question respecting a Home for Epileptics, I beg to say I cannot too strongly recommend that of Maghull, near Liverpool. I have visited there severaltimes. The air is delightful, the house is large, and stands in its own grounds. The patients all appear to be happy, and are well looked after and most kindly treated by Miss Paley, the efficient and thoroughly hospital-trained matron. I fancy the terms are from seven shillings a-week upwards. The home is visited once a-week by Dr. Alexander, one of the leading surgeons in Liverpool, who takes great interest in the place. A doctor resides near the home. All letters should be addressed to Miss Paley, Manor House, Maghull, Liverpool, and she will answer all inquiries as to terms, and forward papers to be filled up, &c.—*A. K.*

There is an incurable ward for women and girls at St. Lucy's Home, Hare Lane, Gloucester, managed by the Clewer Sisters. The ward is very pretty and comfortable, but it may be full. Particulars could be had by writing to the Sister Superior, C. S. J. B.—*M. H. Harrison.*

Apply to Miss Wicksteed, St. John'sRoad, Watford.—*E. T.*

*A Hospital Life Governor* must be aware that his name is required, not for publication, but as a guarantee of good faith. Unless he sends it it is impossible to publish the letter in question.

*One Who Knows.*—If St. Olave's Union is governed in a similar manner to the London Hospital, we can only congratulate the nurses who are privileged to work there.

*Lucy A. D.*—We know nothing save the advertisement.

### Appointments.

[It is requested that successful candidates will send a copy of their applications and testimonials, with date of election, to The Editor, The Lodge, Porchester Square, W.]

Suffolk General Hospital, Bury St. Edmunds.—Miss Harris has been appointed matron of the above hospital. She trained at Addenbrooke's Hospital, Cambridge, and for the last two years has been matron of the Carlisle Fever Hospital. There were 40 candidates.

### Vacancies.

The following vacancies are announced:

**Assistant Superintendent.**

Nottingham Nursing Association; £60.

**Matrons.**

Birmingham Skin and Lock Hospital.

Exeter Eye Infirmary; £40.

**Nurses.**

Alexandra Hospital for Children, Brighton.

Bury St. Edmund's Hospital (night); £20.

Brighton Fever Hospital; £24.

Bournemouth Victoria Home; £25.

Blackheath Nursing Institution.

Brixton Nursing Institute.

Birmingham Hospital for Women; £23.

Borough Hospital, Birkenhead; £25.

Bristol District Nurses' Society.

Chester Deaconess House; £24.

Cumberland Infirmary; £24.

Cheltenham Institution.

Catholic Nurses' Association, Notting Hill, W.

City Hospital, Liverpool; £25.

Croydon Union (night); £22.

Chelsea Infirmary; £18.

Chichester Infirmary (head); £30.

Devizes County Asylum; £25.

Forest Gate District Schools; £20.

General Nursing Institute.

Glasgow Fever Hospital.

Hanwell Ophthalmic School; £26.

Huddersfield Infirmary; £24.

International Institution, Paris; £28.

Jersey Nurses' Institute.

Kent Nursing Institution; £30.

Liverpool Home for Epileptics; £22.

Lowestoft Hospital; £20.

Leicester Fever Hospital; £25.

Mr. Wilson's Institution.

Newcastle Nurses' Home.

Northwood Fever Hospital, Ramsgate; £28.

Nurses' Institute, Southampton.

Ryde Infirmary (night); £25.

St. Saviour's Infirmary; £20.

Sheffield Nurses' Home.

Southampton Fever Hospital; £40.

St. Leonard's, Shoreditch; £20.

St. Helena Home.

Truro Nurses' Home; £25.

Tyrrell Hospital, Ilfracombe; £18.

Westhampnett Union; £22.

Workhouse Infirmary Nursing Association.

**District Nurses.**

Shipley, Yorks.

Hulme Nurses' Home.

Manchester Sick Poor Institute.

Edinburgh Jubilee Institute.

Farnborough, Hants.

**Probationers.**

Bromley Sick Asylum; £16.

Blackheath and Charlton Cottage Hospital.

Bristol Nurses' Training Institution.

Bedford Hospital for Sick Children.

Canterbury Nurses' Institute.

Central Ophthalmic Hospital.

Highgate Convalescent Hospital.

Huddersfield Infirmary; £12.

Hull Infirmary.

Kidderminster Infirmary.

Leeds Nurses' Institution; £14.

Liverpool Cancer and Skin Hospital.

Nurses' Institute, Southampton.

Nurses' Home, Stoke-on-Trent.

St. Helena Home, N.W.

### Amusements and Relaxation.

### SECOND QUARTERLY WORD COMPETITION.

Commenced July 5th, 1890, ends Sept. 27th, 1890.

Three prizes of 15s., 10s., 5s., will be given for the largest number of words derived from the words set for dissection.

Proper names, abbreviations, foreign words, words of less than four letters, and repetitions are barred; plurals, and past and present participles of verbs, are allowed. Nuttall's *Standard* dictionary only to be used.

N.B.—Word dissections must be sent in WEEKLY not later than the first post on Thursday to the *Prize Editor*, 140, Strand, W.C., arranged alphabetically, with correct total affixed.

The word for dissection for this, the FIFTH week of the quarter, being

"APRICOTS."

| Names. | July 24th. | Totals. |
|---|---|---|
| Dulcamara | 45 | 80 |
| Tinie | 50 | 85 |
| Henri | 49 | 83 |
| Agamemnon | 50 | 83 |
| Patience | 49 | 82 |
| Brisbane | — | 28 |
| Nurse Hilda | 45 | 77 |
| Lightowlers | 46 | 76 |
| Jenny Wren | 44 | 71 |
| Rhoda | 40 | 62 |
| Annabel | — | 17 |
| Spare Moments | — | 19 |
| Qu'appelle | 45 | 78 |
| Nurse | 29 | 29 |
| A B C | 32 | 32 |

### Notice to Correspondents.

N.B.—Each paper must be signed by the author with his or her real name and address. A *nom de plume* may be added if the writer does not desire to be referred to by us by his real name. In the case of all prize-winners, however, the real name and address will be published.

Competitors can enter for *all* quarterly competitions, but no competitor may take more than *one first prize* during the year.

